# Do social support and eating family meals together play a role in promoting resilience to bullying and cyberbullying in Scottish school children?

**DOI:** 10.1016/j.ssmph.2019.100485

**Published:** 2019-09-14

**Authors:** Richard J. Shaw, Dorothy B. Currie, Gillian S. Smith, Judith Brown, Daniel J. Smith, Joanna C. Inchley

**Affiliations:** a Institute of Health and Wellbeing, University of Glasgow, Glasgow, UK; b MRC/CSO Social and Public Health Sciences Unit, Institute of Health and Wellbeing, University of Glasgow, UK; c School of Medicine, University of St Andrews, St Andrews, UK

**Keywords:** Cyberbullying, Bullying, Wellbeing, Resilience, Social support

## Abstract

This study investigates if cyberbullying is associated with wellbeing independently of traditional bullying and if social support and eating family meals together promotes resilience by buffering adolescents against the consequences of both types of bullying. Data for 5286 eleven, thirteen and fifteen year olds participating in the cross-sectional 2018 Scottish Health Behaviour in School-aged Children study were analysed. Adolescent self-report measures were used to assess traditional bullying, cyberbullying, classmate and teacher support and frequency of family meals together. Psychological wellbeing was assessed with the 5-item World Health Organization Wellbeing index. Analyses were conducted separately by gender with multilevel models, adjusting for sociodemographic factors. Resilience to bullying and cyberbullying was operationalised using statistical interactions. For both genders, cyberbullying and traditional bullying measures were associated with reduced wellbeing and all social support indicators were associated with increased wellbeing. In models containing both bullying measures, frequent traditional bullying victimisation was associated with a 7.2 (95% CI: 3.4–10.1) reduction in wellbeing score for boys and a 7.2 (95% CI: 4.5–10.0) reduction for girls, while cyberbullying was associated with 10.5 (95% CI: 5.8–15.1) reduction in wellbeing score for boys and 11.1 (95% CI: 6.7–15.5) reduction for girls. For both genders adjusting for classmate support explained away the relationships between traditional bullying and wellbeing, but cyberbullying was associated negatively with wellbeing independent of social support. Only one of 12 interaction tests provided any evidence of resilience. Cyberbullying was associated with a 7.8 (95% CI: 0.2–15.4) reduction in wellbeing score for girls who ate with their family every day, and 17.3 (95% CI: 10.5–24.1) reduction for girls who ate with their families less than weekly. In conclusion, cyberbullying is a strong, albeit rare, threat to adolescent wellbeing. Social support is important for wellbeing, but its ability to buffer adolescents against the consequences of bullying may be limited.

## Highlights

• Cyberbullying is associated with poorer wellbeing independent of bullying.
• Traditional bullying's association with wellbeing is explained by classmate support.
• Little evidence that social support buffers against bullying's adverse consequences.

## 1 Introduction

There are increasing concerns about the effect that cyberbullying has on the mental wellbeing and long term development of adolescents (Arseneault, 2018). Cyberbullying is a comparatively new form of bullying and has been suggested as a possible explanation for rising rates of depression, particularly among adolescent girls (Mojtabai, Olfson, & Han, 2016). However, others dispute whether or not cyberbullying presents an additional threat over and above traditional bullying (Olweus & Limber, 2018; Zych, Ortega-Ruiz, & Del Rey, 2015). Understanding the degree to which cyberbullying influences wellbeing independently from traditional bullying, and whether social support and eating family meals together promote resilience to both forms of bullying, are important considerations for developing anti-bullying strategies in schools and at government level.

### 1.1 Traditional bullying and cyberbullying

Traditional bullying has been defined as purposeful unwanted negative (aggressive) behaviour that is typically repeated towards a victim and occurs when there is a power imbalance favouring the perpetrator (Olweus & Limber, 2018). In cyberbullying, the medium of the internet changes the nature of these interactions between victim and perpetrator(s) (Kowalski, Giumetti, Schroeder, & Lattanner, 2014; Smith et al., 2008). The use of online technology changes power relations: perpetrators can potentially be anonymous, and a single incident of cyberbullying can be disseminated rapidly and persist online, changing the nature of repetition (Gaffney & Farrington, 2018). Cyberbullying is also more difficult to escape because children and young people can be exposed to it in their own homes (Worsley, McIntyre, & Corcoran, 2019). Consequently the impact of cyberbullying could potentially be greater than that of traditional bullying (Hamm et al., 2015).

Prevalence estimates of both bullying and cyberbullying vary widely, but overall the literature suggests that traditional bullying is more common than cyberbullying (Hamm et al., 2015; Modecki, Minchin, Harbaugh, Guerra, & Runions, 2014) and that girls are more likely to be cyberbullied than boys, whereas boys are more likely to be victims of traditional bullying (Hamm et al., 2015; Zych et al., 2015), particularly physical and verbal bullying, but not relational bullying for which the risk of being a victim is higher for girls (Bradshaw, Waasdorp, & Johnson, 2015; Wang, Iannotti, & Nansel, 2009). Systematic reviews and meta-analyses indicate that both forms of bullying correlate strongly (Gini, Card, & Pozzoli, 2018; Kowalski et al., 2014; Modecki et al., 2014). However, there is debate over whether they represent the same thing, with some authors emphasising that they are different phenomena (Antoniadou & Kokkinos, 2015), while others argue it is better to focus on traditional bullying because cyberbullying creates very few new victims (Olweus, 2012; Wolke, Lee, & Guy, 2017).

Adverse consequences of traditional bullying are well established. Immediate consequences for the affected adolescent can include psychological distress, self-harm, academic difficulties and loneliness (Klomek, Sourander, & Elonheimo, 2015; Zych et al., 2015). In addition, long term adult consequences include negative health outcomes such as depression, suicidal thoughts, higher health service usage, and social disadvantages such as difficultly in maintaining employment, marital breakdown and criminality (Klomek et al., 2015; Ttofi, Farrington, Lösel, & Loeber, 2011). Cyberbullying is also associated with a wide range of poor mental health outcomes including suicidal ideation, depression, anxiety, reduced life satisfaction and low self-esteem (Fisher, Gardella, & Teurbe-Tolon, 2016; Hamm et al., 2015; Kowalski et al., 2014; Zych et al., 2015). While there is substantial evidence that cyberbullying influences internalizing and externalizing symptoms independent of traditional bullying (Gini et al., 2018), this is not the case for all outcomes. In particular, research on the relationship between traditional and cyberbullying and measures of positive affect or wellbeing are rare. Those studies that do exist tend to use measures of self-esteem or life satisfaction rather than general wellbeing (Fisher et al., 2016).

### 1.2 Resilience to bullying and cyberbullying

Resilience is the process of positive adaptation in the face of adversity (Masten & Cicchetti, 2016; Masten & Tellegen, 2012). Despite the best efforts of national, local and school-level policies (Arseneault, 2018; Gaffney & Farrington, 2018) bullying remains widespread and it is important to identify factors that might promote resilience to bullying and cyberbullying. In this paper, rather than focusing on the role of protective and risk factors in relation to adolescents’ experiences of traditional bullying and cyberbullying, for which there is already an extensive literature (Kowalski et al., 2014; Zych et al., 2015), we focus on identifying factors which may promote wellbeing and provide additional protective effects in the presence of traditional bullying and cyberbullying, with resilience operationalised using interaction effects (Masten & Cicchetti, 2016; Roosa, 2000).

Different forms of social support may be key factors which promote resilience to traditional and cyberbullying (Masten & Cicchetti, 2016; McDonald et al., 2019). There are three main sources of support family, friends and school personnel (Chu, Saucier, & Hafner, 2010) and they have all been associated with reduced risk of being a victim of bullying (Kowalski et al., 2014; Zych, Farrington, & Ttofi, 2019; Zych et al., 2015) and improved wellbeing (Chu et al., 2010). However, not all sources of support are necessarily equally important. A meta-analysis showed that support from teachers was much more important than support from friends for adolescent wellbeing (Chu et al., 2010). The small number of studies that have investigated sources of support as promotive factors that buffer adolescents against the consequences of experienced bullying (Averdijk, Eisner, & Ribeaud, 2014; Bowes, Maughan, Caspi, Moffitt, & Arseneault, 2010; Davidson & Demaray, 2007; Hodges, Boivin, Vitaro, & Bukowski, 1999; Holt & Espelage, 2007; Prinstein, Boergers, & Vernberg, 2001) or cyberbullying (Elgar et al., 2014; Frison, Subrahmanyam, & Eggermont, 2016; Worsley et al., 2019) have produced inconsistent results. In addition these studies have concentrated on measures of negative affect such as psychological distress, or internalizing and externalizing symptoms, which do not capture positive wellbeing, and consequently results may be constrained by ceiling effects.

In this study we use the teacher support scale to operationalise school personnel support and the classmate support scale to operationalise friend support. Both scales have been developed by the HBSC international network (Inchley, Currie, Cosma, & Samdal, 2018; Torsheim, Wold, & Samdal, 2000). We use a measure of eating family meals together as an indicator of family support. Eating together presents opportunities for young people to discuss social and emotional issues and may help to develop coping strategies (Elgar, Craig, & Trites, 2013) and is strongly related to other measures of family support (Fulkerson et al., 2006). We use “indicators of social support” when we refer to teacher support, classmate support and eating family meals together collectively.

The aim of this study is to investigate whether cyberbullying has an influence on adolescent wellbeing independently of traditional bullying, and to assess how cyberbullying and traditional bullying interact in terms of their relationships with wellbeing. We also investigate the degree to which eating family meals together and support from teachers or classmates is associated with adolescent wellbeing and whether these indicators of social support might buffer the impact of both forms of bullying on wellbeing.

## 2 Methods

Data come from the 2018 Scottish Health Behaviour in School-aged Children (HBSC) study, part of the WHO Collaborative Cross-national HBSC study (Inchley et al., 2018). The Scottish HBSC sample was designed to be nationally representative of 11, 13 and 15 year olds in Scotland. The survey was conducted using classes as the primary sampling unit and all students in the selected class were asked to complete a questionnaire anonymously under exam conditions in the classroom setting. The target population was schoolchildren in the final year of primary school (P7: average age 11.5 years), and in the second (S2: average age 13.5 years) and fourth (S4; average age 15 years) years of secondary school. In total 5286 students from 208 schools participated in the survey. The response rate for classes was 61%, and it is estimated that within those classes 83% of students completed the survey.

### 2.1 Subjective wellbeing

The outcome of the study is subjective wellbeing assessed using the World Health Organization Well-Being Index (WHO-5) (Topp, Østergaard, Søndergaard, & Bech, 2015). The WHO-5 consists of five items (*I have felt cheerful and in good spirits, I have felt calm and relaxed, I have felt active and vigorous, I woke up feeling fresh and rested, and My daily life has been filled with things that interest me*) assessing how the adolescents have been feeling in the last two weeks. The possible responses (*At no time, Some of the time, Less than half of the time, More than half of the time, Most of the time, All the time*) are scored from 0 to 5. The items are added together and then multiplied by 4 to produce a score ranging from 0 (worst possible wellbeing) to 100 (best possible wellbeing). It is a measure of positive affect that has been validated for use in adolescents (de Wit, Pouwer, Gemke, Delemarre-van de Waal, & Snoek, 2007) and also has validity as a screening tool for depression and as a generic measure of wellbeing (Topp et al., 2015).

### 2.2 Traditional bullying and cyberbullying

Traditional bullying was assessed using an adapted version of the Olweus bullying questionnaire (Solberg & Olweus, 2003). Students were given a definition of bullying which emphasized the main characteristics (intentionality, power imbalance and repetition), and were then asked how often they had been bullied at school in the past couple of months. Cyberbullying was assessed after the question on traditional bullying by providing examples of cyberbullying which included sending mean messages, creating websites making fun of a person, or taking inappropriate pictures and posting online without permission, within the last couple of months. Responses for both measures were classified similarly: *Never (I have not been bullied/cyberbullied in past couple of months) Occasional (It has happened once or twice), frequent (2 or 3 times a month/about once a week/several times a week).*

### 2.3 Promotive factors

Three variables were selected that could indicate access to potential sources of support that might promote resilience to bullying and cyberbullying. These were frequency of eating family meals together, classmate support and teacher support.

Eating family meals together was assessed by asking students *“How often do you and your family usually have meals together”* and responses were coded as every day, most days or once a week or less.

Classmate and teacher support were assessed using scales developed by the HBSC international network (Inchley et al., 2018; Torsheim et al., 2000). For classmate support, students were asked their extent of agreement with three statements (*The pupils in my class(es) enjoy being together/Most of the pupils in my class(es) are kind and helpful/Other pupils accept me as I am*) on a five point scale ranging from strongly agree (coded as 4) to strongly disagree (coded as 0). Responses were summed to create a scale ranging from 0 to 12. Similarly teacher support was assessed using three statements (*I feel that my teachers accept me as I am/I feel that my teachers care about me as a person/I feel a lot of trust in my teachers*) and responses were summed to create a scale ranging from 0 to 12.

### 2.4 Sociodemographic factors

Sociodemographic factors included in the study were: family structure (both parent, single parent, step family, other); country of birth (Scotland, Other UK, and Non UK); parental employment (Neither employed, One, Both); and grade and gender specific quintile of the Family Affluence Scale (Currie et al., 2008).

### 2.5 Statistical methodology

Analyses were carried out separately by gender. Survey weights were used to calculate percentages. The main analyses were carried out on complete cases in three stages using multilevel regression using the mixed command from Stata 14.1, with the three levels being pupils, schools and local authority. Following the multilevel models the margins command were used to calculate adjusted averages (Williams, 2012). Likelihood ratio tests were used to test for nonlinearity for the continuous measures and for statistical interactions that might indicate resilience.

In the first stage of analyses we investigated the relationships between wellbeing and the key variables of interest, namely, traditional bullying, cyberbullying, frequency of eating meals together, classmate support and teacher support. We started by presenting bivariate relationships between wellbeing and each of the key measures of interest (model 1), we then adjusted for sociodemographic factors (model 2), before creating a mutually adjusted model (model 3).

In the second stage of analyses we focused on investigating whether cyberbullying had a relationship with wellbeing independent of traditional bullying and then tested for possible interactions.

The third stage of analyses focused on identifying possible promotive factors that might buffer the adolescents against the adverse consequences of bullying and cyberbullying. To do this we tested if models - that included sociodemographic measures, one of traditional bullying or cyberbullying, and one potentially promotive factors - were improved by including an interaction between the bullying term and a promotive factor.

## 3 Results

### 3.1 Descriptives

Sample characteristics are shown in Table 1. Girls were considerably more likely to report they were cyberbullied at least occasionally, but girls were only marginally (p = .062) more likely than boys to report that they were victims of traditional bullying. Boys had higher mean wellbeing scores and student support scores, and were more likely to report eating a family meal together every day. There was no evidence of significant gender differences for teacher support and the socio-demographic factors, apart from family affluence. For all variables there was more missing data for boys than girls.

**Table 1:** Descriptive statistics.

		Boys				Girls			
		n	Weighted %			n	Weighted %	gender difference p value	
**Total**		2581	100.0			2705	100.0		
**Traditional bullying victimisation**									
Never		1634	63.2			1643	61.6	.062	
Occasionally		540	21.4			608	22.5		
Frequent		336	12.5			398	14.1		
Missing		71	2.9			56	1.8		
**Cyber bullying victimisation**									
Never		2138	83.1			2113	78.6	<.001	
Occasionally		254	9.5			415	15.1		
Frequent		113	4.4			126	4.5		
Missing		76	3.0			51	1.9		
**Eat family meals together**									
Once a week or less		613	24.7			611	22.4	.037	
Most Days		995	37.3			1144	42.7		
Every Day		920	36.2			914	33.7		
Missing		53	1.9			36	1.2		
**Class grade**									
P2 (Age 11)		948	33.3			1008	33.3	.921	
S2 (Age 13)		876	33.3			912	33.3		
S4 (Age 15)		757	33.3			785	33.3		
**Family structure**									
Two parents		1555	59.7			1697	62.1	.802	
Lone parent		591	22.9			609	22.8		
Step Family		172	6.8			192	7.2		
Other		103	4.2			105	3.6		
Missing		160	6.5			102	4.3		
**Country of birth**									
Scotland		2289	88.2			2386	88.2	.696	
Other UK		132	5.1			142	5.3		
Non UK		150	6.3			172	6.4		
Missing		10	0.4			5	0.2		
**Parental employment**									
No parent employed		123	4.7			147	5.4	.564	
One parent employed		572	22.5			596	22.0		
Both parents employed		1812	69.7			1896	70.1		
Missing		74	3.1			66	2.5		
**Grade specific quintile of family affluence**									
1st		469	18.0			425	15.7	<.001	
2nd		488	19.0			648	24.2		
3rd		400	15.4			408	14.4		
4th		611	24.0			689	25.9		
5th		477	18.0			439	16.2		
Missing		136	5.8			96	3.7		
Total		2581	100.0			2705	100.0		
	n	Mean	SD	N		Mean	SD		
WHO5 Wellbeing	2476	59.6	22.02	2632		55.4	23.4	<.001	
Student support Scale	2521	8.09	2.23	2666		7.72	2.36	<.001	
Teacher support scale	2.533	8.61	2.78	2678		8.73	2.67	.114	

**Fig. 1:**
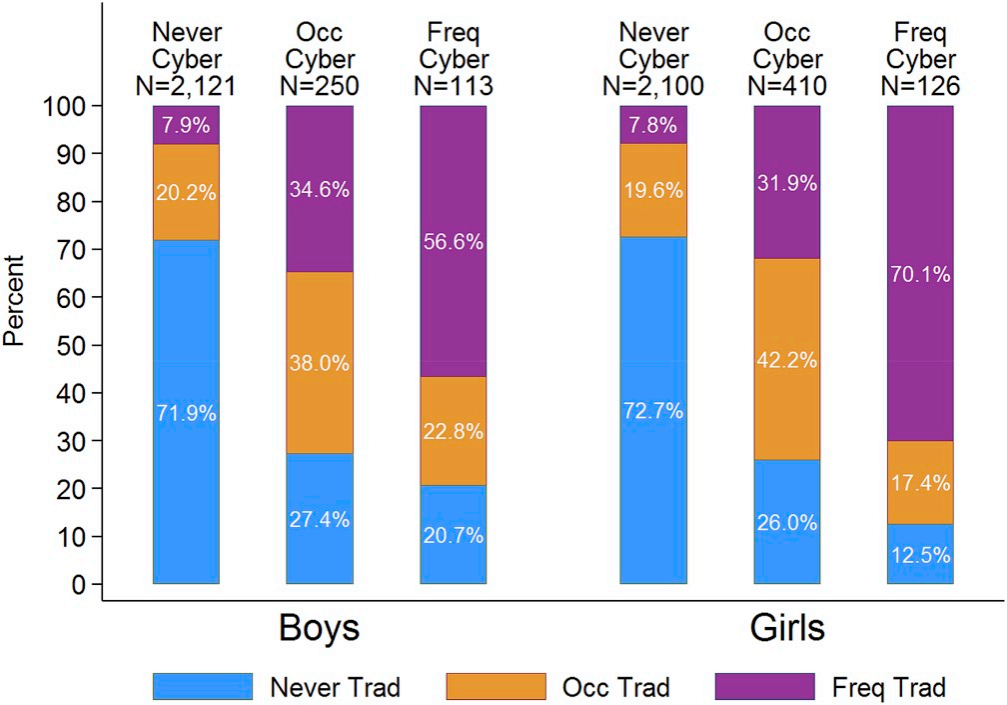
Percentage of adolescents who were victims of traditional bullying by cyberbullying victimisation status and gender.

The percentage of adolescents who were victims of traditional bullying by cyberbullying victimisation status and gender is shown in Fig. 1. This illustrates that there was a very strong relationship between bullying and cyberbullying. Only a relatively small percentage (7.9% of boys and 7.8% of girls) of those who were not cyberbullied were frequently traditionally bullied. Conversely, among those who had been frequently cyberbullied, only 20.1% of boys and 12.5% girls had not been traditionally bullied.

### 3.2 Influence of bullying and indicators of support on wellbeing

**Figure 2:**
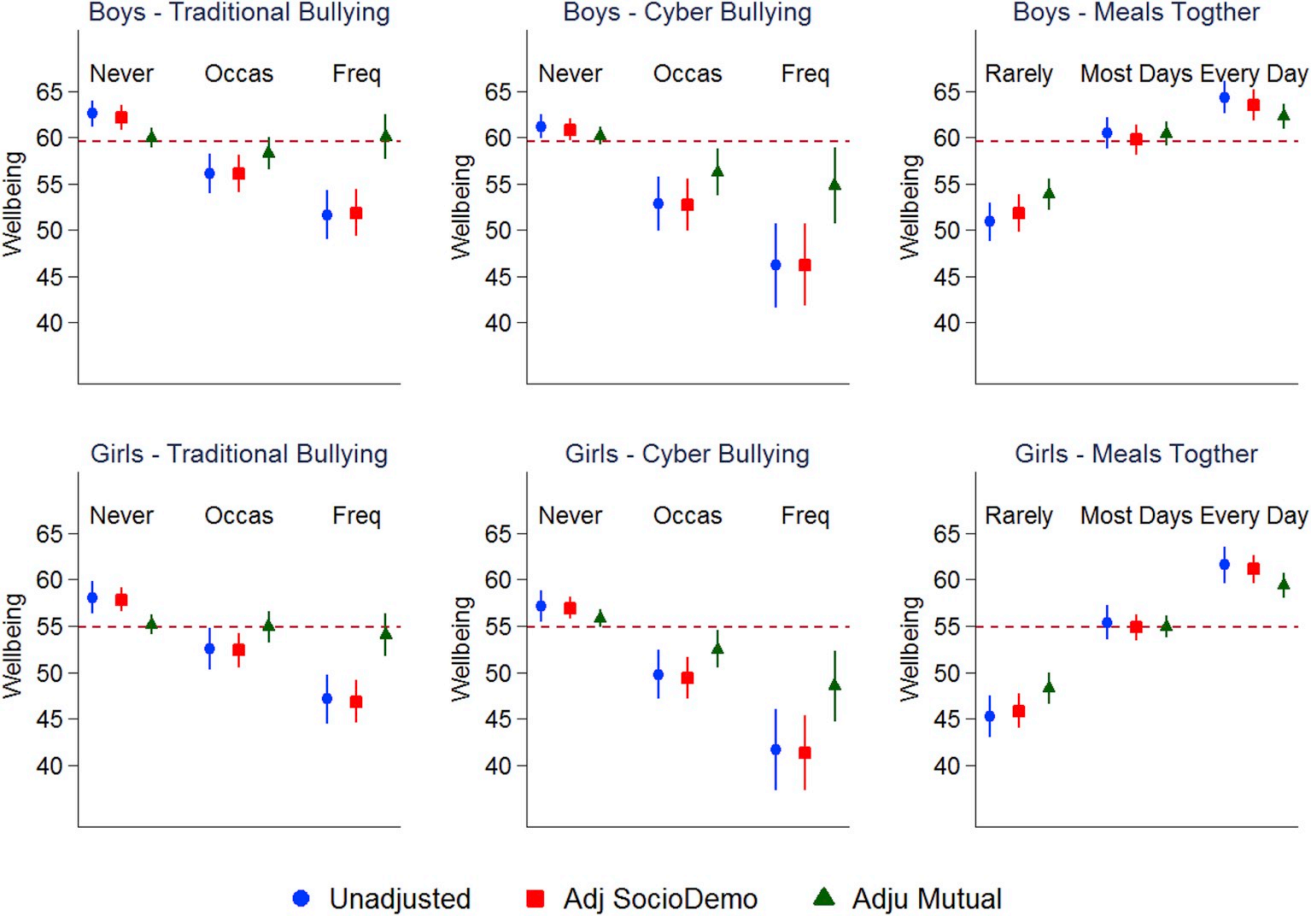
Mean wellbeing (WHO-5 score) by traditional bullying victimisation, cyberbullying victimisation and frequency of eating meals together for boys and girls in unadjusted, adjusting for sociodemographic variables^1^ and mutually adjusted models^2^. Dashed redline indicates genders specific mean. 1. Adjusted for grade, family structure, country of birth, parental employment, and family affluence. 2. As 1 plus adjustment for traditional bullying victimisation, cyberbullying victimisation, frequency of eating family meals together, classmate support and teacher support.

**Fig. 3:**
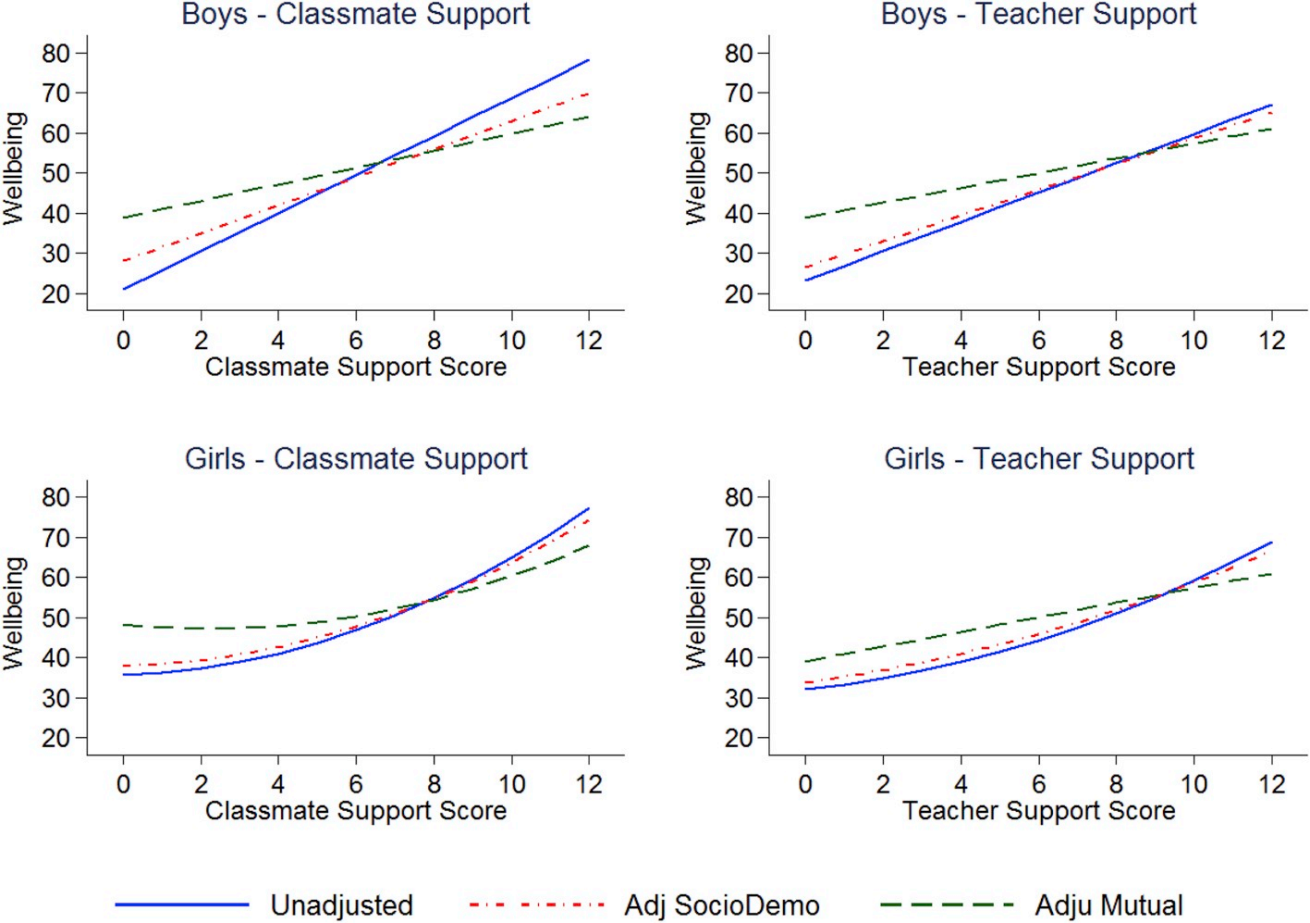
Mean wellbeing by classmate and teacher support for boys and girls in unadjusted, adjusting for sociodemographic variables^1^ and mutually adjusted models^2^. 1. Adjusted for grade, family structure, country of birth, parental employment, and family affluence. 2. As 1 plus adjustment for traditional bullying victimisation, cyberbullying victimisation, frequency of eating family meals together, classmate support and teacher support.

We present predicted wellbeing (WHO-5 score) means by each of the main independent variables of interest: traditional bullying, cyberbullying, frequency of family meals (Fig. 2) and classmate support and teacher support (Fig. 3). The conditional coefficients are presented in Appendix Table 1. These are from unadjusted models, models adjusting for sociodemographic factors (which includes grade, family structure, country of birth, parental employment, and family affluence), and mutually adjusted models including traditional bullying, cyberbullying, frequency of family meals together, classmate and teacher support. In both unadjusted and adjusted models, all key variables of interest show strong relationships. For example the regression coefficients for being a frequent victim of traditional bullying after adjusting for sociodemographic factors are −10.3 (95% CI: -13.0 to −7.6) for boys and −11.0 (95% CI: -13.5 to −8.5) for girls. All the key variables of interest, except traditional bullying, show strong associations with wellbeing even after mutual adjustment. For example the regression coefficients for frequently being a victim of cyberbullying are −5.4 (95% CI: -9.6 to −1.2) for boys and −7.3 (95% CI -11.3 to −3.4) for girls. In additional analyses (not presented here), we found that classmate support alone explained nearly all the difference between the coefficients for frequent traditional bullying from the models adjusting for sociodemographic factors and the same coefficient for the mutually adjusted models. Using a model that adjusts for sociodemographic factors as a base, adding classmate support reduces the coefficient for frequent bullying victimisation by 86% in boys and 73% in girls. For girls, classmate support and teacher support show nonlinear relationships with wellbeing (see Fig. 3).

### 3.3 Interactions between bullying and cyberbullying

**Fig. 4:**
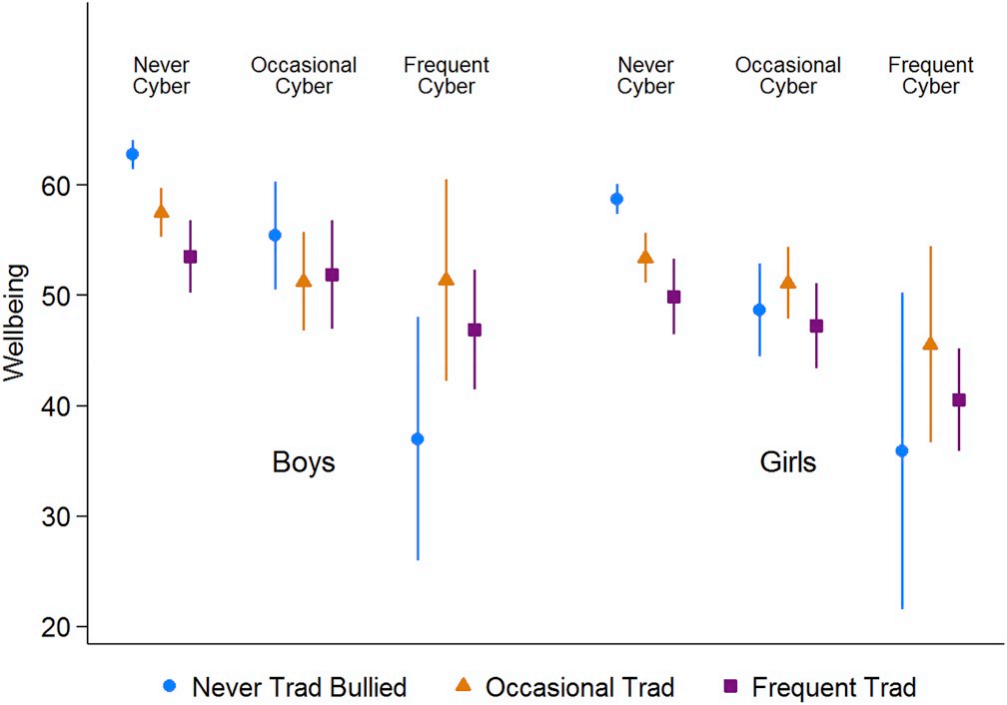
Mean wellbeing from models showing an interaction between traditional bullying and cyberbullying for boys and girls in models adjusting for sociodemographic factors.1.Adjusted for grade, family structure, country of birth, parental employment, and family affluence.

Focusing on whether traditional bullying and cyberbullying have independent relationships with wellbeing in models adjusting for sociodemographic variables, the indicators of support were omitted as they could at least partially mediate the relationships between the bullying measures and wellbeing (Du, DeGuisto, Albright, & Alrehaili, 2018). We found not only evidence of independent relationships between the bullying measures and wellbeing (see appendix Table 2), but also evidence of an interaction between the bullying measures for both boys (p = .0243) and girls (p = .0302) as shown in Fig. 4. For both genders the results would suggest that bullying and cyberbullying broadly have an independent relationships with wellbeing, with the exception that a small group of adolescents (8 boys and 21 girls, who have been frequent victims of cyberbullying but not traditional bullying, have very low wellbeing scores (Fig. 4).

### 3.4 Testing for interactions that might indicate resilience

**Fig. 5:**
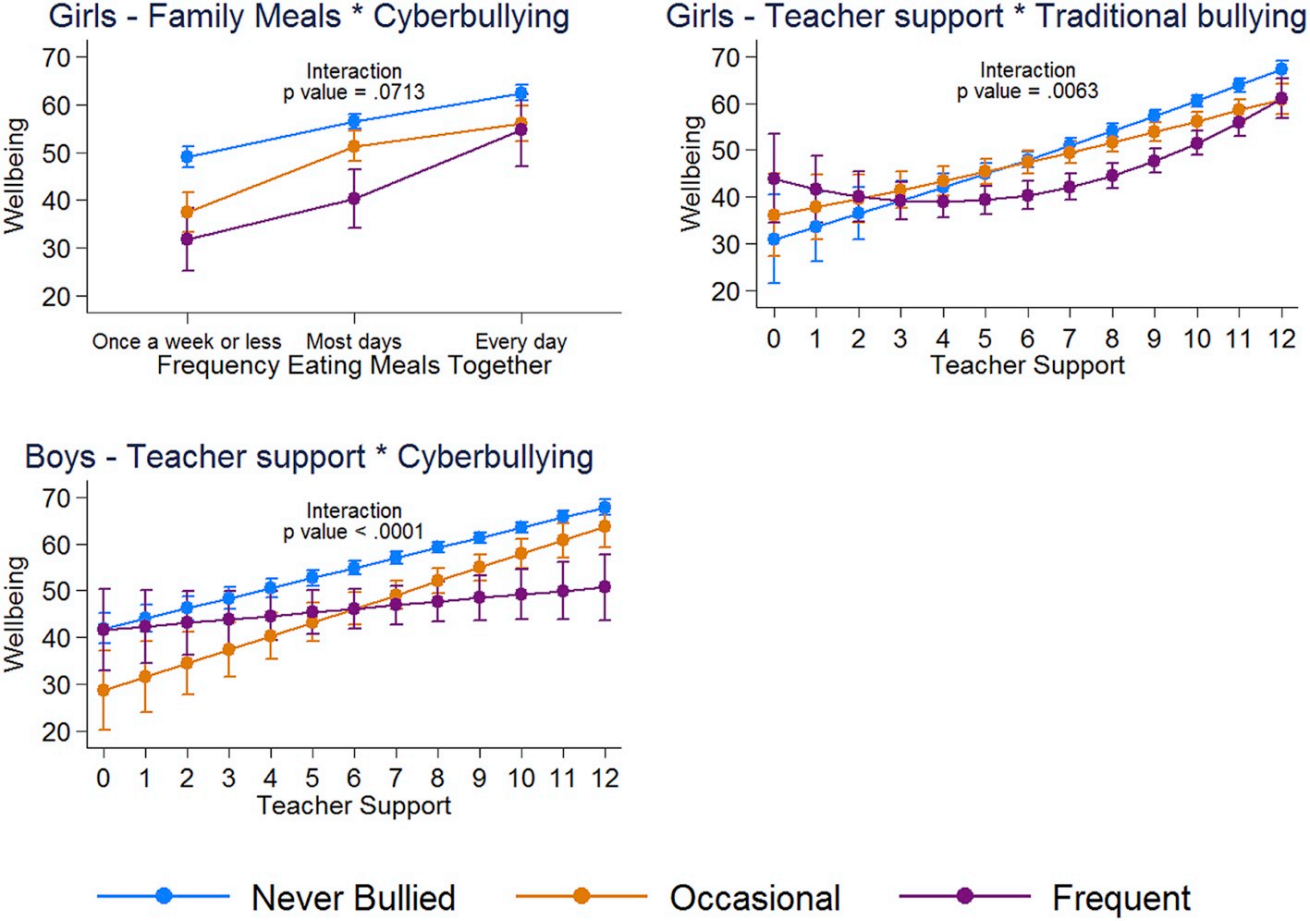
Mean wellbeing from models with significant interaction terms (p < .10) between measures of bullying and indicators of support adjusting for sociodemographic factors.^1^ 1.Adjusted for grade, family structure, country of birth, parental employment, and family affluence.

We investigated whether there was evidence of resilience by testing for statistical interactions between in turn, one of traditional bullying or cyberbullying, and one of the potentially promotive factors – eating family meals together, classmate support and teacher support. Out of 12 tests we found only three where there might plausibly be some evidence of statistical interactions and these are shown in Fig. 5. For girls there was some evidence of an interaction between cyberbullying and family meals such that cyberbullying was associated with a stronger reduction in wellbeing among those who ate family meals together less than once a week (17.3, 95% CI: 10.5 to 24.1), compared with girls who ate meals with their family every day (7.8, 95% CI: 0.2 to 15.4) see Fig. 5. We also found interactions between teacher support and traditional bullying for girls, and teacher support and cyberbullying for boys, however, the pattern of interactions are not consistent with those of resilience (Luthar, Cicchetti, & Becker, 2000), and may indicate reverse causality or measurement issues at the extremes.

## 4 Discussion

### 4.1 Summary

For both boys and girls we found that cyberbullying and traditional bullying are adverse risks for poorer wellbeing, and that the relationship between traditional bullying and wellbeing was mostly explained by peer relationships as measured by classmate support. This was despite the fact that where cyberbullying occurs it was almost always alongside traditional bullying. The indicators of social support are all associated independently with improved wellbeing, however, the evidence that social support helps promote resilience to either bullying's or cyberbullying's negative influences on wellbeing was limited.

Our results add to the existing literature by showing that bullying and cyberbullying are negatively associated with wellbeing, a measure of positive affect, in addition to the well-established associations with internalizing and externalizing symptoms and other negative outcomes (Klomek et al., 2015; Ttofi et al., 2011; Zych et al., 2015). We reinforce the evidence that cyberbullying has a relationship with mental wellbeing independently of traditional bullying (Gini et al., 2018), while at the same time providing support for the notions that cyberbullying rarely creates additional victims (Olweus, 2012; Wolke et al., 2017) and that girls are more likely to be cyberbullied than boys (Hamm et al., 2015; Zych et al., 2015). However, there were no gender differences for traditional bullying.

Our results for the social support indicators are consistent with the existing literature that social support promotes better wellbeing (Paniagua, Moreno, Rivera, & Ramos, 2019). However, we found little evidence to support the idea that the indicators of support buffer adolescents against the adverse effects of bullying and cyberbullying, and hence promote resilience to bullying. This is not necessarily out of line with the pre-existing literature. Of those older studies that do find some evidence of interactions that might indicate resilience, many carried out multiple statistical tests between different types of bullying and support and only found significant interactions in one or two cases, which might be expected by chance (Davidson & Demaray, 2007; Hodges et al., 1999; Holt & Espelage, 2007; Prinstein et al., 2001). More recent studies have shown greater consistency, for example Bowes et al. (2010) found multiple sources of support were associated with resilience against bullying's possible consequences for emotional and behavioural problems. Similarly, Elgar et al. (2014) in a large (n > 18,000) study found that associations between cyberbullying and internalizing and externalizing symptoms, and substance abuse, were much stronger for students who reported never having had family meals together than for students who had family meals together more regularly. Among these more recent studies those that included measures of cyberbullying (Elgar et al., 2014; Frison et al., 2016; Worsley et al., 2019) tend to find more associations that indicate resilience than those that focus on traditional bullying (Averdijk et al., 2014). Our study is consistent with the idea that eating family meals together might promote some degree of resilience to cyberbullying. It is possible that our results provide more limited evidence of resilience compared to previous studies because we assess wellbeing using the WHO-5. WH0-5 is able to capture aspects of positive affect in addition to negative symptoms and may be less restricted by ceiling effects. Social support may reduce bullying's association with pathological behaviour and states, but may not mitigate against bullying's consequences for more positive aspects of wellbeing. Alternatively, the ability of school-related social support to protect against bullying may be specific to school-related outcomes rather than more general wellbeing measures.

### 4.2 Strengths and weaknesses

The study's strengths include analysis of a large general population sample in Scotland and using established survey instruments, but there are a number of limitations which need to be considered. We operationalise bullying using single item measures which may be less sensitive than multi-item measures (Kowalski et al., 2014). We also only carried out analyses using a single outcome measure, and the measures of traditional bullying, cyberbullying and indicators of social support may not have the same relationships or interact with each other in the same way for other outcomes. We also acknowledge that while we used eating family meals together as an indicator of social support, it does not measure support directly and is merely an indicator of the opportunity for adolescents to communicate with their family and obtain support. Eating family meals together is also likely to relate to other aspects of the home environment including parenting practices and monitoring which could explain any relationships it has (Elgar et al., 2014). In preliminary analyses family and friend support measures derived from the Multidimensional Scale of Perceived Social Support (Zimet, Dahlem, Zimet, & Farley, 1988) and used in the HBSC study had a nonlinear relationship, requiring multiple polynomial terms, with not only wellbeing (results available from the authors on request), but also with other health related outcomes and the eating family meals together variables. We have not presented these results because they were inconsistent with theory and may reflect certain methodological limitations which require further investigation. Additionally, the polynomial terms made it very difficult to interpret the statistical interactions testing for resilience. For the sake of parsimony we present models adjusting for sociodemographic factors and mutual adjustment, but theoretically the optimal causal model for each risk factor might lie somewhere between the two. However, given the constraints of using cross-sectional data and the inability to test the directionality of associations, for example teacher support may not only be a cause of higher levels of adolescent's wellbeing, but also a response to adolescents who have poorer wellbeing and are distressed, we deemed this an acceptable compromise.

### 4.3 Implications

Cyberbullying appears to represent an additional threat to wellbeing independently of traditional bullying, despite cyberbullying victimisation being rare in the absence of traditionally bullying. Additionally, classmate support explains the relationship between traditional bullying (but not cyberbullying) and wellbeing. Both of these results are consistent with the idea that cyberbullying and traditional bullying are carried out in different contexts, and this is also consistent with the results of Du et al.’s (2018) study. This is perhaps not surprising given that the traditional bullying measure used in HBSC is specific to school-based bullying while the cyberbullying measure may encompass a much wider social context. The finding that cyberbullying has a stronger association with poorer wellbeing, among those who have not otherwise been bullied face-to-face, raises interesting questions. It is possible that adolescents who have been bullied previously may have developed maladaptive coping strategies, such as social isolation, which may help them avoid traditionally bullying but still have detrimental consequences for wellbeing. Alternatively, cyberbullying in the absence of traditional bullying might represent a less common but potentially more serious threat to wellbeing, perhaps because cyberbullying can be more covert and victims are less likely to seek help (Dooley, Gradinger, Strohmeier, Cross, & Spiel, 2012). Future research could use longitudinal data or qualitative methods to investigate the circumstances in which cyberbullying has occurred in the absence of traditional bullying.

Eating family meals together and social support from peers and teachers are clearly important for adolescent health, with young people reporting higher levels of support also reporting higher levels of wellbeing. However, while there is some evidence that eating family meals together may mitigate the impact of bullying on wellbeing, our findings indicate these effects are small, suggesting that absence of bullying and presence of good support are both required to promote adolescent wellbeing.

## 5 Conclusions

Cyberbullying has a relationship with poorer wellbeing independent of traditional bullying. The relationship between traditional bullying and wellbeing appears to be largely explained by classmate support. While cyberbullying rarely occurs in the absence of traditional bullying, it should be considered an additional threat to healthy development. Although eating family meals together and support from classmates and teachers are all associated with higher wellbeing, their ability to buffer adolescents against the adverse consequences of traditional bullying or cyberbullying appear to be limited.

## Ethical statement

Ethical approval for the survey was granted by the University of St Andrews School of Medicine Research Ethics Committee.

## Declarations of interest

None.

## Figures and Tables

**Table:** 

	Model 1: Unadjusted			Model 2: Adjusted Socio demog^1^			Model3: Mutually Adjusted^2^		
	Coef	95% CI		Coef	95% CI		Coef	95% CI	
		Lower	Upper		Lower	Upper		Lower	Upper
*Boys*									
**Traditional Bullying**									
Never									
Occasionally	−6.45	−8.69	−4.22	−6.03	−8.21	−3.85	−1.64	−3.63	0.35
Frequent	−10.89	−13.62	−8.16	−10.31	−12.97	−7.64	0.16	−2.50	2.83

**Cyber bullying Victimisation**									
Never									
Occasionally	−8.32	−11.31	−5.33	−8.11	−11.01	−5.21	−3.98	−6.66	−1.31
Frequent	−14.98	−19.52	−10.44	−14.55	−18.97	−10.13	−5.41	−9.59	−1.23

**Family Meals**									
Once a week or less									
Most Days	9.54	7.21	11.87	7.91	5.61	10.20	6.54	4.49	8.59
Every Day	13.41	11.04	15.78	11.62	9.27	13.97	8.39	6.28	10.49

**Student Support**									
Linear term	4.77	4.40	5.14	4.39	4.00	4.77	3.53	3.11	3.96

**Teacher Support**									
Linear term	2.77	2.45	3.09	2.42	2.10	2.75	1.11	0.78	1.43

*Girls*									
**Traditional Bullying**									
Occasionally	−5.54	−7.69	−3.39	−5.46	−7.52	−3.40	−0.30	−2.26	1.66
Frequent	−10.94	−13.53	−8.37	−11.03	−13.52	−8.85	−1.10	−3.76	1.56

**Cyber bullying Victimisation**									
Not									
Occasionally	−7.37	−9.79	−4.94	−7.59	−9.91	−5.27	−3.31	−5.55	−1.08
Frequent	−15.50	−19.73	11.26	−15.65	−19.71	−11.59	−7.33	−11.30	−3.35

**Family Meals**									
Once a week or less									
Most Days	10.17	7.92	12.41	8.99	6.81	11.17	6.66	4.67	8.65
Every Day	16.37	14.02	18.73	15.29	13.01	17.57	11.09	8.98	13.2

**Student Support**									
Linear term	0.24	−1.26	1.74	0.23	−1.22	1.67	−0.95	−2.37	0.46
Quadratic term	0.227	0.16	0.37	0.23	0.13	0.33	0.22	0.12	0.31

**Teacher Support**									
Linear term	0.99	−0.46	2.45	1.30	−0.11	2.71	1.83	1.48	2.18
Quadratic term	0.17	0.08	0.26	0.11	0.03	0.21			

1. Adjusted for grade, family structure, country of birth, parental employment, and family affluence.

2. As 1 plus adjustment for traditional bullying victimisation, cyberbullying victimisation, frequency of eating family meals together, classmate support and teacher support.

**Table:** 

	Boys			Girls		
	Coefficients	Confidence interval		Coefficients	Confidence interval	
		Lower	Upper		Lower	Upper
**Traditional Bullying victimisation**						
Never						
Occasionally	−4.94	−7.15	−2.74	−3.94	−6.08	−1.80
Frequent	−7.24	−10.11	−3.37	−7.24	−10.02	−4.45

**Cyber bullying Victimisation**						
Never						
Occasionally	−5.65	−8.64	−2.66	−5.05	−7.53	−2.57
Frequent	−10.48	−15.13	−5.82	−11.06	−15.47	−6.65

1. Adjusted for grade, family structure, country of birth, parental employment, family affluence, traditional bullying and cyberbullying.
